# Supplementary figures and images for: A Cell-Type–Resolved Meta-Analysis Reveals Glial DNA Methylation Changes Associated with Aging and Alzheimer’s Disease
Source: bioRxiv. 2026 May 6:2026.05.04.722662. Preprint. [Version 1] doi: 10.64898/2026.05.04.722662 (PMC13174515; doi:10.64898/2026.05.04.722662)

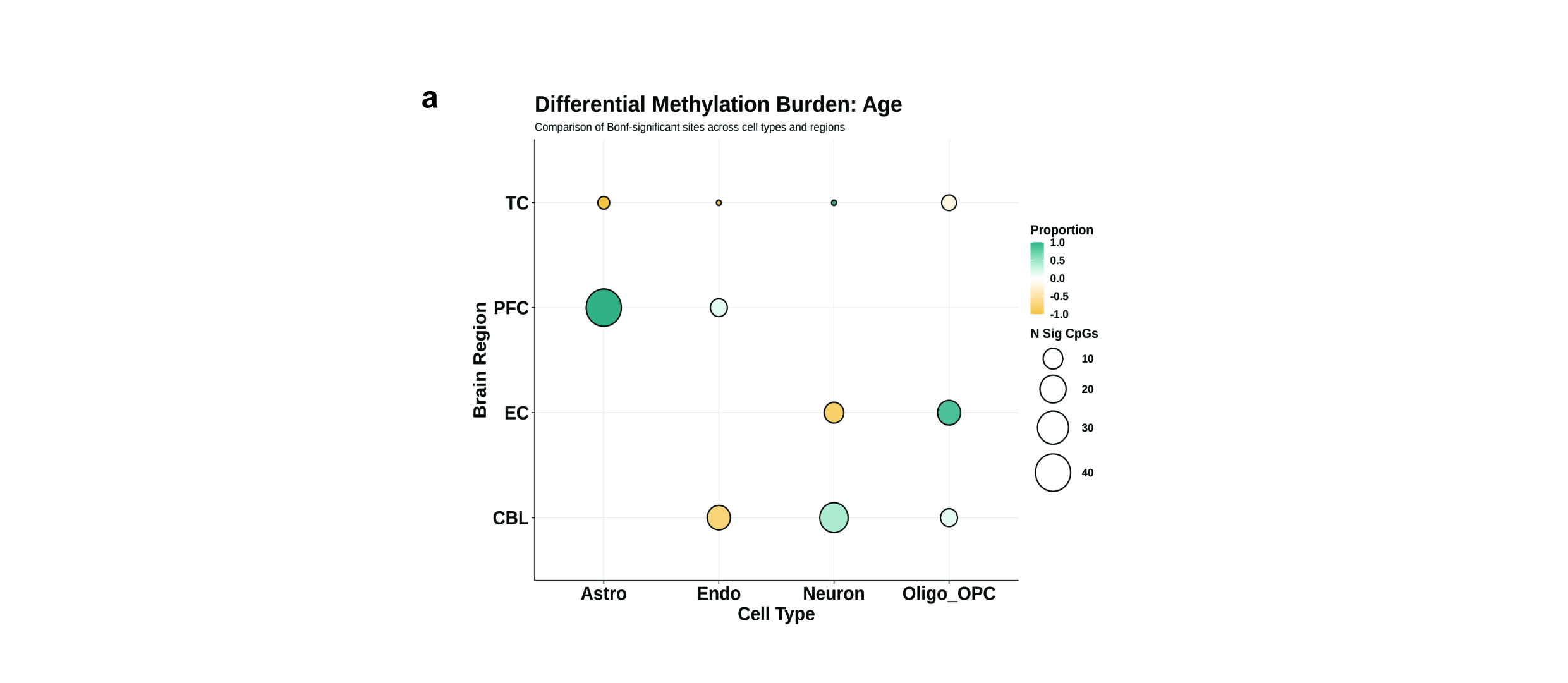

Supplement: Supplement 2 [file media-2.tif]

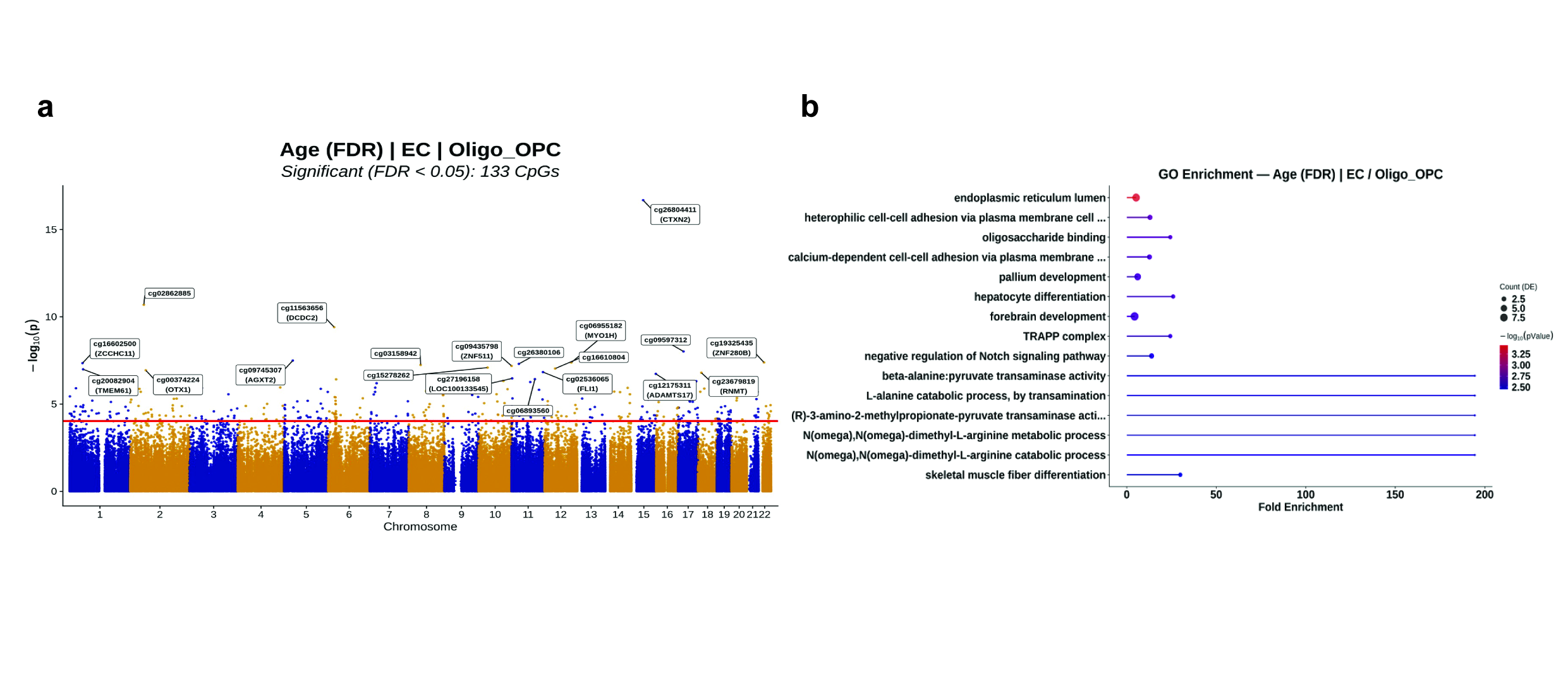

Supplement: Supplement 3 [file media-3.tif]

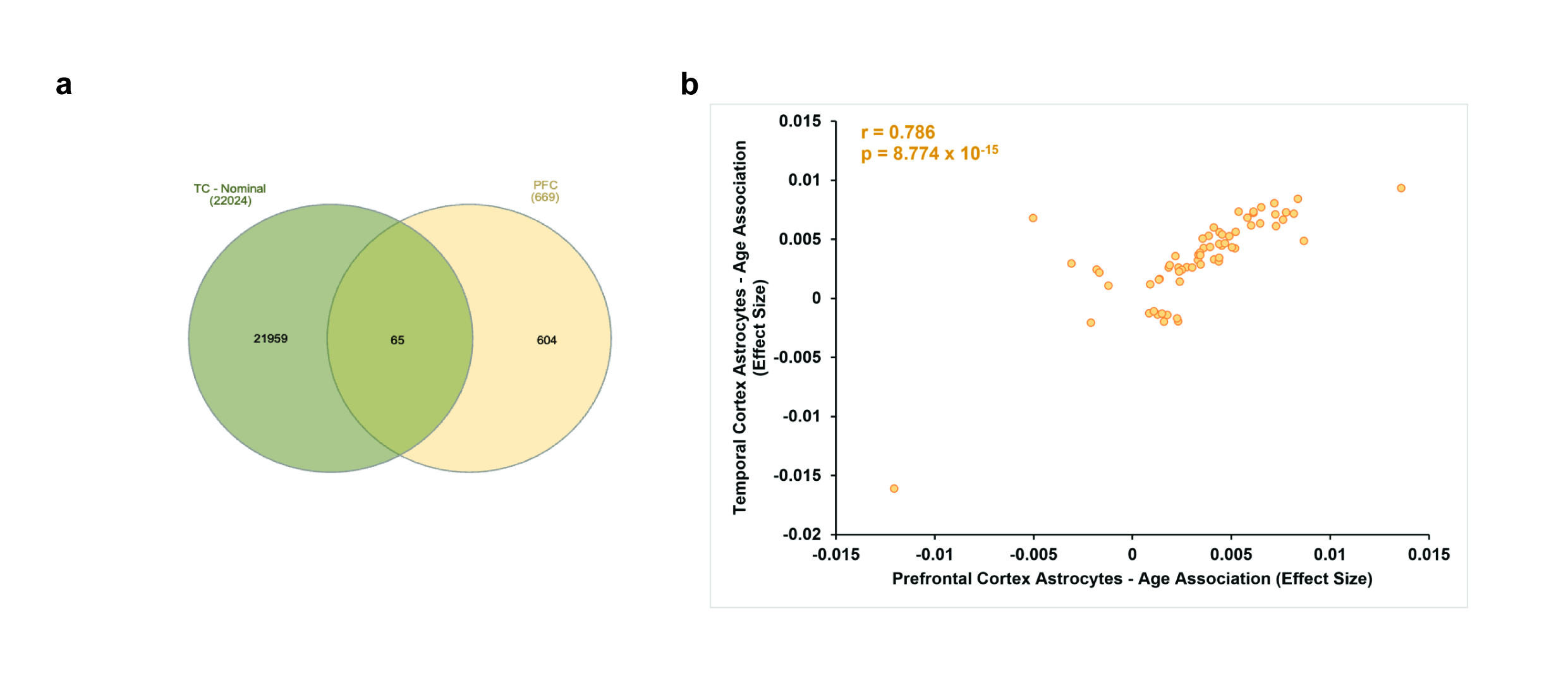

Supplement: Supplement 4 [file media-4.tif]

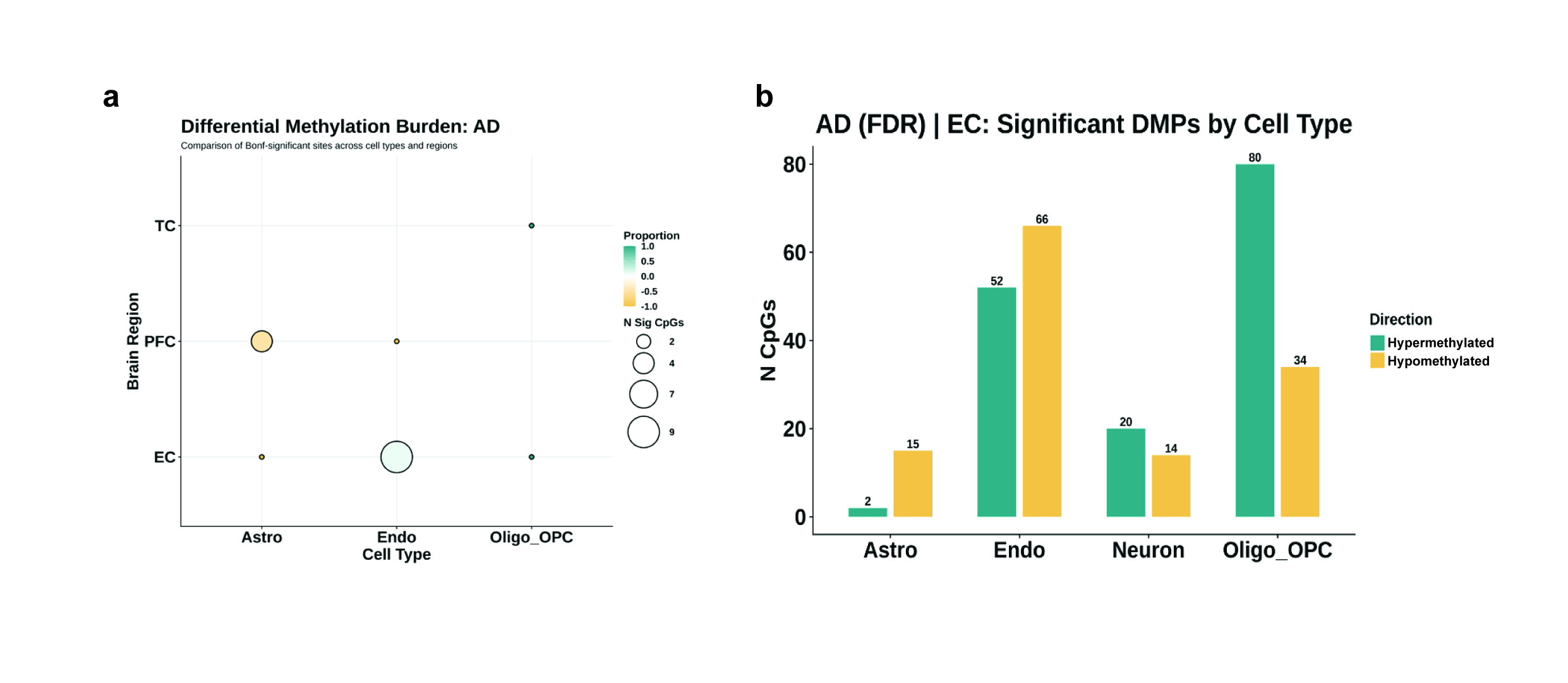

Supplement: Supplement 5 [file media-5.tif]

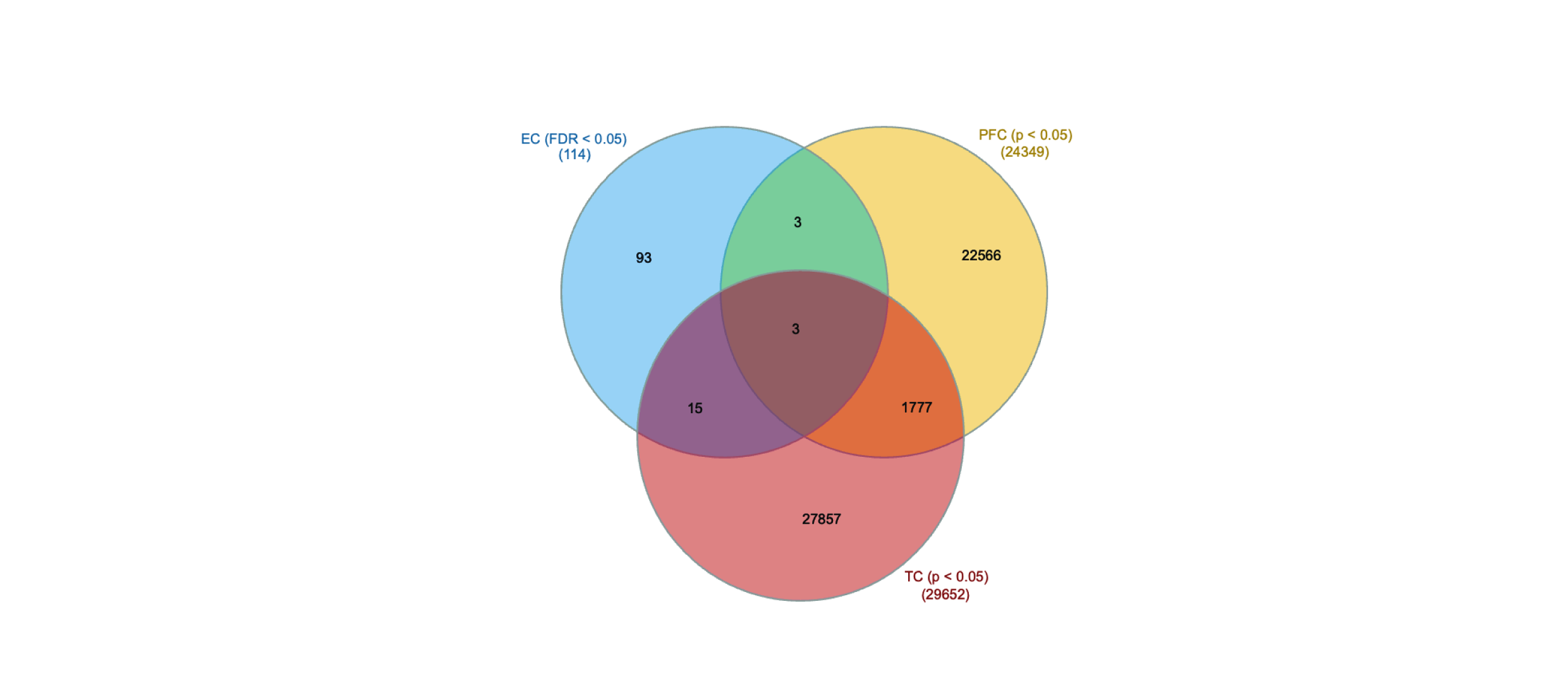

Supplement: Supplement 6 [file media-6.tif]

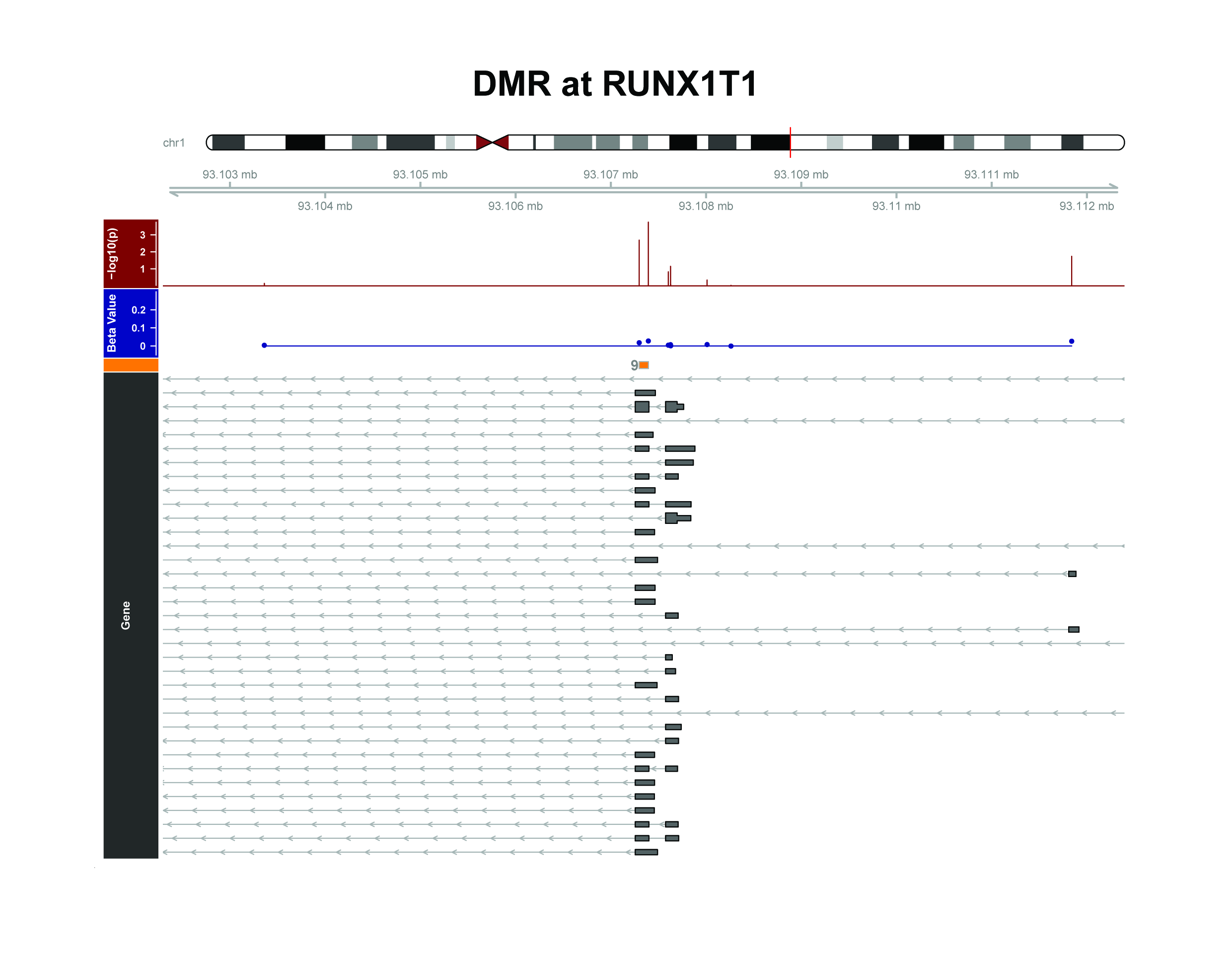

Supplement: Supplement 7 [file media-7.tif]
